# Shame Memory’s Impact on Depression among Junior Middle School Students: A Moderated Mediation Model

**DOI:** 10.3390/bs13100802

**Published:** 2023-09-27

**Authors:** Xinxin Yu, Yijing Pan, Jiaojun Ouyang, Peizhen Sun

**Affiliations:** 1 Department of Psychology, School of Education, Guangxi Normal University, Guilin 541006, China; 2 Department of Psychology, School of Education Science, Jiangsu Normal University, Xuzhou 221116, China

**Keywords:** shame memory, depression, self-criticism, emotional management, middle school students

## Abstract

(1) Objective: While recent studies have shed light on the effects of shame memories on mental well-being, there is still limited understanding of the underlying mechanisms linking shame memories and depression. Based on the biopsychosocial model and cognitive therapy theory, this study examined the association between shame memory and depression and the indirect role of emotion management and self-criticism. (2) Methods: A total of 1004 junior high school students were measured with the Center for Epidemiologic Studies Depression Scale, the event impact scale, the self-criticism scale of the depressive experiences questionnaire, and the emotional intelligence scale. (3) Results: Shame memory had a significant positive correlation with depression. The relationship between shame memory and depression was partially mediated by self-criticism. Emotional management was found to regulate the latter half of the mediating effect of shame memory on depression. (4) Conclusions: Self-criticism plays a mediating role in the relationship between shame memory and depression, as well as emotion management plays a moderating role between self-criticism and depression. This perspective will contribute to the growing body of knowledge about the impact of shame memories on depression among junior high school children but also offers a feasible plan for follow-up intervention.

## 1. Introduction

More than 350 million individuals worldwide suffer from depression, with a patient growth rate of roughly 18% over the past decade, according to data released by the World Health Organization (WHO) in 2019. Moreover, there is about one million depression-related suicide each year. It has been estimated that currently up to 100 million people in China are plagued by depression, with an incidence rate of between 3 and 5 percent, compared to 3.1% globally. Depression accounts for 40 percent of the annual 280,000 suicides annually in China. The Blue Book of the National Mental Health Progress Report of China (2019–2020), published in 2021, states that nearly 24.6% of adolescents suffer from various degrees of depression, of which 7.4% are severely depressed, and the student population is the main sufferer of adolescent depression, accounting for up to 40% [1]. Also, the nation and all sectors of society are paying more and more attention to teen depression. Adolescents who have experienced intense feelings of shame and self-criticism during their teenage years are more susceptible to depression. It is crucial for teachers, parents, and relevant researchers to be highly concerned about middle school students who fall into this category.

Shame memories pose serious harm to adolescents’ mental health and increase their risk of developing depression. While recent studies have shed light on the effects of shame memories on mental well-being [2,3,4], there is still limited understanding of the underlying mechanisms linking shame memories and depression. This study aims to investigate the mediating role of self-criticism in the relationship between shame memory and depression, as well as the moderating role of emotion management between self-criticism and depression, based on the Biopsychosocial model [5] and cognitive therapy theory [6]. Through this investigation, the paper will uncover the internal mechanisms of the effect of shame memory on depression.

### 1.1. Shame Memories and Depression

Shame memories are traumatic memories that can be easily acquired when an individual perceives himself as inadequate and worthless in situations such as criticism, rejection, or abuse. These memories give rise to negative self-beliefs and psychologically painful experiences [7]. Gilbert (1998) [5] held that shame memory increases the inclination towards shame, which also strengthens the personal experience of shame in diverse settings, according to his biopsychosocial model. This model emphasizes the significance of social standing and cultural values in determining whether social groups are accepted or rejected [8]. As a result, sensitive individuals to shame memories often exhibit a high level of avoidance in social situations, a source of depression because of the self’s perception of being flawed and inferior to others in the group. They also worry about their perception of themselves from others. Shame is a sensitive component in the development of depression and has a symbiotic relationship with depression [4] as people in shame frequently experience sentiments of self-denial and doubt like “I have flaws, I am horrible, I am a mistake, and I made a mistake” [9]. Based on this, this study proposes hypothesis 1: shame memory of junior high school students serves as a positive predictor of depression.

### 1.2. The Mediating Role of Self-Criticism between Shame Memory and Depression

Shame memory and self-criticism have long been linked to psychopathology, particularly depression [10]. Shame puts individuals in a meditative and self-critical style, rendering them vulnerable to various difficulties [11]. According to the theory of emotional attribution, people with high vulnerability to shame tend to internalize, stabilize, and generally self-attribute to negative occurrences. As a result, they experience a sense of shame at negative events, which would further exacerbate self-criticism and depression [12]. A recent study also noted that self-criticism, which may take various forms and serve different purposes, is closely tied to shame memory. The first is that the “Inadequate Self” is typically used to rectify acts when one feels unhappy or upset about them for the mistakes one makes; the second is that when one’s ego is judged to be abhorrent, flawed, and worthless, it is typically intended to harm and attack oneself [13].

Blatt (1974) [14] systematically discussed the close connection between self-criticism and many mental health issues in his research on the relationship between personality and depression. He also highlighted the possibility that self-criticism contributes to individual dysfunction and impedes the development of personality traits. In Beck’s (1983) [6] cognitive therapy theory, he postulated that one of the causes of depression is a high level of personal self-criticism. Busch (2009) [15] proposed that certain depressed people may excessively deprecate or criticize themselves in an attempt to manage their anger levels, which may result in a more severe depression. According to research, those engaging in self-criticism are more likely to experience introjective depression following a failure. Self-criticism is also a crucial personality trait that develops during socialization and is inextricably linked to depression [16]. When faced with negative feelings or failure-related experiences, adolescents who are overly self-critical and blame themselves are more likely to experience depression than their peers [17]. Based on these findings, this study proposes hypothesis 2: self-criticism in junior high school students acts as a mediator between depression and shame memory.

### 1.3. The Moderating Role of Emotion Management between Self-Criticism and Depression

Emotional management refers to how individuals express their emotions when dealing with events that trigger emotional changes [18]. According to Ellis’ ABC theory of emotion, emotional suffering is frequently brought on by irrational beliefs, which result in emotional illnesses if not addressed. On the other hand, shame is a trigger of a variety of mental health issues [3], including anxiety, depression, bulimia, and other maladaptive symptoms [19]. Shame memory also has traumatic aspects [20]. Moreover, studies have demonstrated a connection between childhood memories of being threatened and current experiences of self-criticism and depression [21], and self-criticism functions as a complete mediator between shame memory and depression [13]. In addition, individuals with a higher level of emotional management tend to be more positive and better equipped to cope with negative feelings [22] and improve their emotional management ability to be more psychologically healthy and complete their personalities [23]. In conclusion, this study put forth hypothesis 3: emotion management of junior high school students can moderate the relationship between self-criticism and depression.

**Figure 1:**
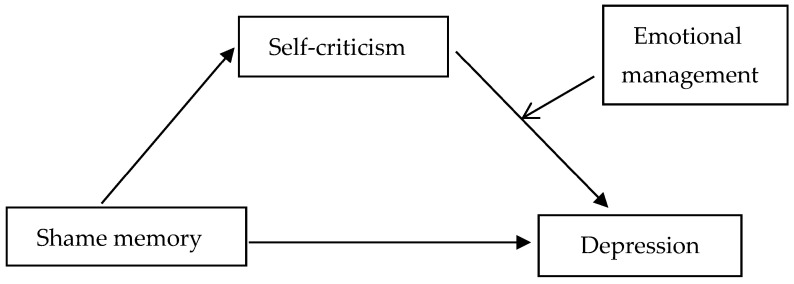
Hypothetical model.

In conclusion, this study builds a moderated mediation model (see Figure 1) to explore the effects of shame memory on depression and the underlying mechanism of self-criticism and emotion management. As such, the study will provide a new theoretical perspective on the mechanism that contributes to depression, offer a reference for reducing depression among junior high school students, and promote their healthy development.

## 2. Method

### 2.1. Procedure

With the help of the cluster sampling method, the paper test was carried out with the school class as the unit. Questionnaires were administered to obtain informed consent from the principals of the target schools as well as from the participants and their parents. After consent was obtained, trained research assistants would enter the target schools to collect data. During the data collection process, the research assistants would clarify any questions participants had about the survey design.

### 2.2. Participants

A total of 1100 junior high school students were selected from five junior high schools in Guangxi and Hunan as the research objects. After removing the invalid questions with excessive missing values, questions with the same option, and answers with inconsistent facts, 1004 valid questionnaires were obtained, with an efficiency rate of 91.3%. Among them were 529 boys, accounting for 52.7%, and 475 girls, representing 47.3%. The sample also comprised 418 first-year students, representing 41.6%, and 586 senior-year students, accounting for 58.4%. The participants were from various backgrounds, with 223 urban students (22.2%), 224 county students (22.3%), and 557 rural students (55.5%). The age range is 11–16 years old (M = 13.6 years old, SD = 0.74 years old).

### 2.3. Measures

#### 2.3.1. Depression

The Center for Epidemiologic Studies Depression Scale (CES-D) is used to assess the frequency of depressive symptoms in the last week. There are 20 items that cover four domains: depression, positive emotions, physical symptoms, and interpersonal relationships. Four items, namely 4, 8, 12, and 16, are reverse scoring [24]. Each item is rated on a 4-point Likert scale from “rarely or none of the time (less than 1 day)” to “most or all of the time (5–7 days)” 0–3 points are, respectively, scored, and the total score is 0–60 points. A higher total score indicates a higher frequency of depressive symptoms (Cronbach’s alpha = 0.89).

#### 2.3.2. Shame Memory

A Chinese version of the Impact of Event Scale (IES-R; Weiss, 1997) [25] contained 22 items, including three dimensions: avoidance, intrusion, and hyperarousal. Each item is rated on a 5-point scale, from 0 (not at all) to 4 (extremely), and a higher total score represents a greater impact of the event on the individual. The questionnaire is used in this study to measure the level of shame memory among the subjects, and a higher score signifies a higher level of shame memory. This study revealed high internal consistency (0.92).

#### 2.3.3. Self-Criticism

The translated version of the self-criticism scale of the depressive experiences questionnaire (DEQ-SC) contained 15 items [26]. Each item is rated on a 7-point scale, from 1 (strongly disagree) to 7 (strongly agree). Higher scores on this scale indicate a greater inclination towards self-criticism (Cronbach’s alpha = 0.87).

#### 2.3.4. Emotional Intelligence

A Chinese version of the emotional intelligence scale (WLEIS) [27] contained 16 items, including self-emotional assessment and others’ emotional assessment, management, and utilization. Each item is rated on a 7-point scale, from 1 (totally disagree) to 7 (totally agree). The higher the score, the greater the level of emotional intelligence. This study uses the emotional management dimension questionnaire. A higher score on the questionnaire indicates a higher level of emotional management (internal consistency = 0.86).

## 3. Result

### 3.1. Common Method Bias Test

As all the research data were obtained through self-reported subjects, the study employed the Harman one-factor common method deviation test to assess the possibility of common method bias. The results show 13 factors with characteristic roots greater than 1, and the variance interpretation rate of the first factor is 23.05%, below the critical value of 40%. Therefore, the study is largely free of common method bias.

### 3.2. Descriptive Statistics and Correlation Analysis

Table 1 shows the mean value, standard deviation, and Pearson correlation coefficient for the main variables in the current study. Shame memory showed a positive correlation with self-criticism and depression while displaying a negative correlation with emotion management. Self-criticism also demonstrated a positive correlation with depression and a negative correlation with emotion management. A significant negative correlation was observed between emotion management and depression.

### 3.3. The Mediating Effect Test

The correlations among the four variables—shame memory, self-criticism, emotion management, and depression—were statistically significant, satisfying the premise of the mediating effect test. The mediating effect test was conducted using Model 4 (simple mediating model) in the SPSS PROCESS v4.0 plug-in, with gender and age as control variables. The aim is to test the mediating effect of self-criticism between shame memory and depression in junior high school students. The results are presented in Table 2.

**Figure 2:**
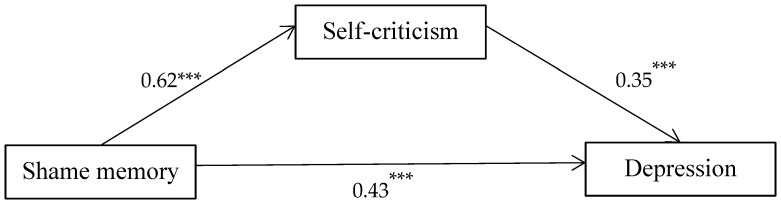
Output model of self-criticism mediation. *** *p* < 0.001.

The mediating analysis showed that shame memory could significantly positively predict depression when controlling for gender and age (β = 0.64, t = 27.05, *p* < 0.001). Furthermore, self-criticism was included as the mediating variable (β = 0.62, t = 25.21, *p* < 0.001), self-criticism was a significant positive predictor of depression (β = 0.35, t = 12.19, *p* < 0.001), and shame memory remained a significant positive predictor of depression (β = 0.43, t = 15.06, *p* < 0.001). Hypothesis 1 is tested (Figure 2).

The Bootstrap procedure (5000-sample) was used to conduct further testing of the intermediary effect value. The results showed that none of the effect values contained 0 within the Bootstrap 95% confidence interval (Table 3), indicating that self-criticism played a significant intermediary role in the impact of shame memory on depression, and the intermediary effect accounted for 33.54% of the total effect. This shows that shame memory not only has a direct impact on depression but also predicts it through the intermediary role of self-criticism.

### 3.4. Moderated Mediating Effect Test

To explore the role of emotion management on the mediating model, the SPSS PROCESS 3.3 plug-in was used to further examine the moderating effect of emotion management on self-criticism and depression. The moderated mediation model in this study was tested according to model 14 of the PROCESS plug-in in the SPSS macro compiled by Hayes. All variables have been standardized and gender and age are taken as control variables in the model test. In the first step, shame memory was the independent variable and depression was the dependent variable for statistical analysis. In the second step, the interaction terms of shame memory, self-criticism, and emotion management, were taken as independent variables. The results are shown in Table 4.

The results showed that shame memory positively predicted self-criticism (β = 0.62, t = 25.21, *p* < 0.001). Shame memory significantly positively predicted depressive mood (β = 0.41, t = 15.02, *p* < 0.001), self-criticism significantly positively predicted depressive mood (β = 0.33, t = 11.89, *p* < 0.001), and emotion management significantly negatively predicted depressive mood (β = −0.16, t = −7.46, *p* < 0.001). The interaction term of emotion management and self-criticism predicted depression significantly in junior middle school students (β = 0.06, t = 3.16, *p* < 0.01), and emotion management significantly moderated the second half of the mediating effect of shame memory on depression. So, hypothesis 3 is true.

**Figure 3:**
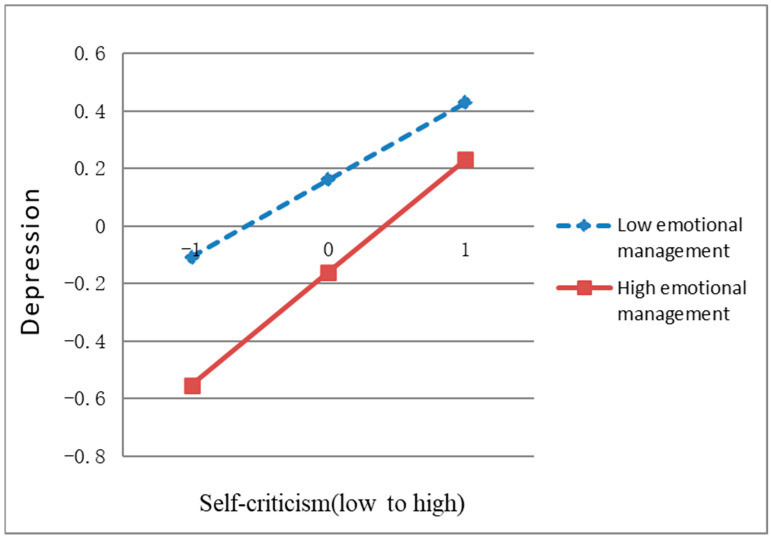
The moderating effect of emotion management on the association between shame memory and depression.

It has been tested that the interaction term of emotion management and self-criticism has a significant predictive effect on junior middle school students’ depressive emotions. To further understand the essence of this interaction, a simple slope test is needed to analyze the moderating effect of emotion management further. To form the high emotion management group, one standard deviation is added to the mean of emotion management. Conversely, to create the low emotion management group, one standard deviation is subtracted from the mean of emotion management. The simple slope test results are shown in Figure 3.

As shown in Figure 3, junior high school students with higher emotion management levels tend to enjoy lower depression than junior high school students with lower emotion management levels. Emotion management plays a protective role to some extent. However, for junior high school students with low emotion management levels, self-criticism has a significant positive predictive effect on depression (*b_simple_* = 0.27, SE = 0.03, *p* < 0.001, 95%CI [0.20, 0.34]). The higher the level of self-criticism, the higher the depressive mood of junior high school students. For junior high school students with higher emotion management levels, self-criticism still had a significant positive predictive effect on depressive mood (*b_simple_* = 0.39, SE = 0.03, *p* < 0.001, 95%CI [0.33, 0.46]).

The Bootstrap method (5000 samples) was used to test the moderated mediation effect, and the results are shown in Table 5.

## 4. Discussion

### 4.1. Shame Memory and Depression

According to the study’s findings, there is a significant positive correlation between depression and memories of shame. This finding supports hypothesis 1 and is consistent with the findings of earlier research [4,28]. Shame autobiographical memory [29,30] explains the memory of shame experiences. When we experience shame during social interactions and anticipate negative treatment from others, it can leave a lasting emotional impact and become a basis for negative self-evaluation. Thus, shame events are often stored in autobiographical memory, affecting one’s sense of self. There is evidence that childhood and adolescent shame memories can shape personal identity, and serve as turning points in life stories and reference points for other meaningful attributions of events [31,32]. All these findings support the centrality of the event theory [29,30], according to which memories of traumatic or negative emotional events can become central to an individual’s life story and identity. The higher the centrality of negative or traumatic events, the higher the post-traumatic stress, depression, anxiety, fragmentation, and poor physical health. Xiao Zhu and Sun Ying et al. [33] explored the relationship between individual shame and depression among college students. They found that individual shame had a direct positive predictive effect on depression, indicating that shame memory is an important predictor of depression.

### 4.2. The Mediating Role of Self-Criticism between Shame Memory and Depression

This research supports hypothesis 2 by showing that shame memories can predict depression in junior high school students both directly and indirectly through the mediation of self-criticism. First, the above results are consistent with the diathesis–stress model derived from depression [34] and the dynamic interaction model of depression vulnerability [35]. The diathesis–stress model believes that individual personality vulnerability and external pressure will jointly lead to depression. According to the dynamic interaction model of depression susceptibility, depression is the result of the interaction of cognitive quality (such as personality, attribution style, self-consciousness, and coping style) and stress events. In the diathesis–stress model, when some external emergency stressors stimulate the susceptible physique, the stressors will have a superimposed effect on the basis of the degree of vulnerability, thus generating a psychological load on the individual. When the total load the individual bears exceeds the threshold, the following physiological and psychological symptoms will appear [36]. Secondly, self-criticism, a personality prone to depression, has a very close tie with depression. When it interacts with the external pressure events related to individuals, it is easy to cause depression to individuals. That is to say, high self-criticism poses a high risk of depression. At the same time, a large number of empirical studies have proved the vulnerability of self-criticism personality to depression. The level of self-criticism can effectively predict depression and positively correlate with depressive symptoms [37,38,39]. Moreover, self-criticism mediates the link between negative memories in early childhood and depression [8]. In the theory of emotional attribution [40], shame is the result of specific attribution of failure. Lewis (1974) [41] first proposed the concept of shame vulnerability, which means that some individuals are always more likely to experience shame when facing negative events. These individuals are called shame-prone individuals. At the same time, the theory of emotional attribution focuses on the relationship between specific negative self-concepts and shame emotion. It proposes that shame emotion can ensure that individuals comply with rules and norms. Still, because this negative cognition points to the whole self, it will significantly impact individual self-esteem and is more likely to have negative consequences on mental health [42]. Tracy and Roberts [43] pointed out in the self-awareness emotional attribution model that an individual’s internal, stable, uncontrollable, and global self-attribution to emotional events would cause shame. Richter et al. [21] also believed that the shameful memory of childhood threatened feelings were related to current self-criticism and depression. Later studies also confirmed that self-criticism functions as an important mediator in the relationship between shame memory and depression [44]. In the research results of Santos et al. [13], self-criticism is a complete mediator between shame memory and depression.

### 4.3. The Moderating Effect of Emotion Management on Self-Criticism and Depression

This study found that emotion management significantly moderates shame memory and depression, supporting hypothesis 3. Emotion regulation moderates shame memory through self-criticism in the second half of junior high school students’ depression pathway but has no moderating effect on the direct pathway.

Emotion is an integral part of human spiritual activities and an external expression of human experience [45]. According to the ABC theory of emotion proposed by the famous American psychologist Ellis, the negative emotions of individuals are caused by the incomplete understanding and evaluation of stressors [46]. Different thoughts will lead to various emotional and behavioral reactions, and different beliefs will lead to different positive and negative beliefs. Ellis believes that people often have unreasonable beliefs that cause them emotional distress. If these irrational beliefs exist for long, they will cause serious adverse consequences such as emotional disorders. Many studies have shown that psychological pressure caused by emotions is easy to cause mental illness [47,48], and the improvement of emotion management can effectively reduce the prevalence of depression [49], and help individuals deal with various adverse emotions, such as anxiety, depression, fear, etc. [50]. Emotion management (EM) is usually associated with emotional intelligence (EI). Emotion management can effectively deal with anxiety and impulses by controlling emotions and persevering in the face of setbacks. It allows individuals to express their emotions, transform negative emotions, and enhance positive emotions in a timely manner [51]. The reason why emotion management ability can play an alleviating role in the relationship bbetween self-criticism and depression may be that individuals with high emotion management ability tend to think rationally when they encounter negative events that have an impact on them under the same circumstances through reflection, re-evaluation, and re-correction of their own thoughts to help individuals in a more reasonable, more consistent situation to look at the problem and get rid of negative emotions on their own influence and interference. The research of Mayer et al. [52] shows that the change in mood will prompt individuals to make more choices and avoid immurement in bad emotions. Specifically, emotion management can enable individuals to accurately understand their true feelings, overcome impulses, delay gratification, regulate emotions, avoid excessive frustration that impairs thinking, empathize with others, understand them sincerely, motivate themselves to overcome setbacks and cultivate hope for the future [53]. The studies of Guo Shan [54] and Liu Jing [55] have shown that psychological intervention of emotion management can significantly relieve patients’ negative emotions and improve their anxiety and depression. Therefore, enhancing adolescents’ emotional management level can reduce the severity of depression [56]. The cognitive theory of depression suggests that individuals believe in the potential long-term persistence of the present frustration dilemma in the future, leading to depression and despair [57]. Suppose the basis for despair is the irrational belief that negative events cannot be solved or changed. In that case, guiding adolescents towards positive thinking can provide the power of hope, which is likely to cushion depression in the face of adversity. The regulating effect of emotion management provides side confirmation and new ideas for depression prevention and intervention among junior high school students. Developing the intervention process related to emotion management or incorporating a psychological course on emotion management suitable for junior high school students into mental health education is conducive to training junior high school students to actively cope with negative stressful events. This can act as a buffer against the adverse effects on individuals and the possible development of depression.

## 5. Limitations and Contributions

This study has the following limitations. First, due to this study’s cross-sectional design, it is impossible to explore the causal relationship among shame memory, self-criticism, emotion management and depression. As a result, future studies must investigate the causal relationship between relevant variables by means of longitudinal design or experiment. Secondly, questionnaire data in this study were obtained by self-assessment, which may have a common method bias. Future research must comprehensively collect data from individuals, peers, parents, teachers and other sources and check the stability of results in various measurement methods. However, the samples in this study come from two different regions, and the possibility of the results being extended to other samples remains to be tested. In the future, we should focus on extensive sample data from all over the country to further test the universality of the results. Despite the above limitations, it also boasts the following contributions. From a theoretical point of view, this study enriched previous studies by revealing the mediating role of self-criticism and the moderating role of emotion management. It deepened the understanding of the mechanism of shame memory’s influence on depression. From the perspective of practice, the discussion of the formation mechanism of depression in junior high school students has a profound practical significance for the design and intervention programs to reduce depression in junior high school students. First, the mediating role of self-criticism suggests to school educators that reducing individual self-criticism helps reduce individual depression. Secondly, the regulating effect of emotion management indicates that intervention programs should focus on individuals with low emotion management levels. Schools can help individuals improve their emotion management level through group counseling.

## 6. Conclusions

The following findings were observed: (1) shame memory had a significant positive correlation with depression; (2) the relationship between shame memory and depression was partially mediated by self-criticism; and (3) emotional management was found to regulate the latter half of the mediating effect of shame memory on depression.

## Figures and Tables

**Table 1:** Describe statistics and correlation.

Variable	M	SD	1	2	3	4
1. Shame memory	1.34	0.79	-			
2. Self-criticism	3.92	1.13	0.632 **	-		
3. Emotional management	4.14	1.50	−0.155 **	−0.159 **	-	
4. Depression	0.90	0.54	0.658 **	0.632 **	−0.289 **	-

** *p* < 0.01 (2-tailed).

**Table 2:** Testing the mediation effect of self-criticism on depression.

Variable	Depression		Self-Criticism		Depression	
	*β*	*t*	*β*	*t*	*β*	*t*
Gender	−0.22	−4.71 ***	−0.18	3.74 ***	−0.16	−3.58 ***
Age	0.05	1.45	−0.00	−0.01	0.05	1.56
Shame memory	0.64	27.05 ***	0.62	25.21 ***	0.43	15.06 ***
Self-criticism					0.35	12.19 ***
*R* ^2^	0.67		0.64		0.72	
*F*	267.88 ***		228.94 ***		267.69 ***	

*** *p* < 0.001 (2-tailed).

**Table 3:** Mediating effect test and 95% confidence intervals of the bootstrap for bias correction.

Effects	Paths	Effect Size	Boot SE	95%CI		Estimate
				LLCI	ULCI	
Direct effect	Shame memory → Depression	0.43	0.03	0.37	0.48	66.46%
Indirect effect	Shame memory → Self-criticism → Depression	0.22	0.02	0.18	0.25	33.54%
Total effects		0.64	0.02	0.60	0.69	

**Table 4:** Moderated mediating effect test (*n* = 1004).

Control Variables	Variable					
	Model 1 (Self-Criticism)			Model 2 (Depression)		
	*β*	*SE*	*t*	*β*	*SE*	*t*
Gender	−0.18	0.05	−0.59	0.14	0.04	−3.29 ***
Age	0.00	0.03	3.74 ***	0.05	0.03	1.68
Shame memory	0.62	0.02	25.21 ***	0.41	0.03	15.02 ***
Self-criticism				0.33	0.03	11.89 ***
Emotion management				−0.16	0.02	−7.46 ***
Self-criticism × emotion management				0.06	0.02	3.16 **
*R* ^2^	0.64			0.74		
*F*	228.94 ***			201.93 ***		

*** *p* < 0.001. ** *p* < 0.010.

**Table 5:** The mediating effect of attribution frustration on social exclusion and depression during different cognitive reappraisals.

Emotional Management	Boot Indirect Effect (*β*)	Boot SE	95%CI	
			Boot LLCI	Boot ULCI
Low level (M − SD)	0.17	0.02	0.12	0.21
M	0.21	0.02	0.17	0.24
High level (M + 1SD)	0.24	0.02	0.20	0.28

## Data Availability

The data that support the findings of this study are available from the corresponding author upon reasonable request.
